# Engineered titania nanomaterials in advanced clinical applications

**DOI:** 10.3762/bjnano.13.15

**Published:** 2022-02-14

**Authors:** Padmavati Sahare, Paulina Govea Alvarez, Juan Manual Sanchez Yanez, Juan Gabriel Luna Bárcenas, Samik Chakraborty, Sujay Paul, Miriam Estevez

**Affiliations:** 1 Centre of Applied Physics and Advanced Technologies (CFATA), National Autonomous University of Mexico, Queretaro, Mexico; 2 Instituto de Investigaciones Químico Biológicas, Universidad Michoacana de San Nicolas de Hidalgo, Morelia, Mexico; 3 Grupo de Investigación de Biomateriales, Ciencia de los Materiales, Cinvestav, Querétaro, Mexico; 4 Division of Nephrology, Boston Children’s Hospital, Harvard Medical School, Boston, MA 02115, USA; 5 Tecnologico de Monterrey, School of Engineering and Sciences, Campus Queretaro, Av. Epigmenio Gonzalez, No. 500 Fracc. San Pablo, CP 76130 Querétaro, Mexico

**Keywords:** clinical application, nanostructures, physicochemical, theranostics, titanium dioxide (TiO_2_)

## Abstract

Significant advancement in the field of nanotechnology has raised the possibility of applying potent engineered biocompatible nanomaterials within biological systems for theranostic purposes. Titanium dioxide (titanium(IV) oxide/titania/TiO_2_) has garnered considerable attention as one of the most extensively studied metal oxides in clinical applications. Owing to the unique properties of titania, such as photocatalytic activity, excellent biocompatibility, corrosion resistance, and low toxicity, titania nanomaterials have revolutionized therapeutic approaches. Additionally, titania provides an exceptional choice for developing innovative medical devices and the integration of functional moieties that can modulate the biological responses. Thus, the current review aims to present a comprehensive and up-to-date overview of TiO_2_-based nanotherapeutics and the corresponding future challenges.

## Introduction

**Figure 1:**
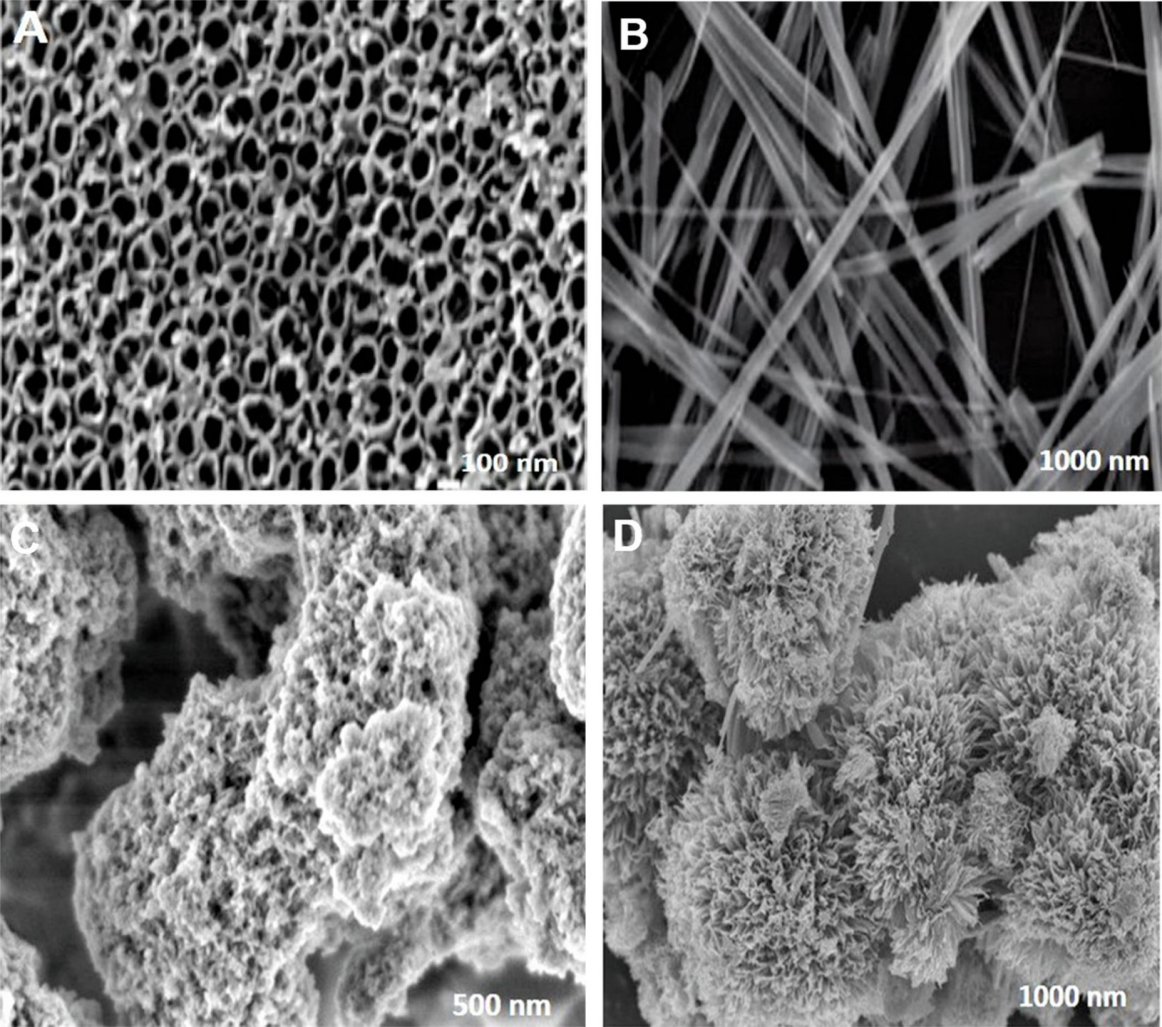
SEM images of titania structures. (A) Nanotubes. (Figure 1A was adapted with permission from [5], Copyright 2005 American Chemical Society. This content is not subject to CC BY 4.0.) (B) Nanobelts. (Figure 1B was adapted with permission from [6], Copyright 2010 American Chemical Society. This content is not subject to CC BY 4.0.) (C) Mesostructure (unpublished image) and (D) nanoflowers. (Figure 1D was adapted with permission from [7], Copyright 2018 American Chemical Society. This content is not subject to CC BY 4.0.)

Nanomaterials can be described as any organic, inorganic, or organometallic material whose chemical, physical, and/or electrical properties change as a function of the size and shape of the material. Nanomaterials are designed at the atomic or molecular level, and most of the therapeutic nanoparticles (nps) are usually between 10 and 100 nm in size so that they can circulate freely through the circulatory system and can penetrate tissues. Recently, TiO_2_ has received substantial recognition as one of the most extensively studied inorganic metal oxides in clinical research due to its unique nanosized features, intrinsic properties, biocompatibility, and low toxicity [1]. TiO_2_ nanomaterials can be applied in a host of applications, including biomedical, optical, electronic, mechanical, and chemical fields, amongst other scenarios [2]. The application of titania nanomaterials in the pharmaceutical field has brought revolutionary changes by providing new and innovative medical solutions. About 1300 nanomaterials are currently available worldwide, with TiO_2_ being the second most abundantly used material in our day-to-day life. Advancement in nanotechnology has resulted in the fabrication of different forms of TiO_2_ nanostructures, such as nanotubes , nanobelts, mesostructured, nanoflowers, including many more as displayed in the SEM image of Figure 1 [3]. Moreover, TiO_2_ has recently been approved for use in food and drug products by the American Food and Drug Administration (FDA) [4].

The first clinical application of nanoscale TiO_2_ was reported by Rehman [8], who used the photodynamic properties of TiO_2_ for killing HeLa cancer cells. The hydrophobic nature of photosensitizers commonly used in photodynamic therapy led to selectivity and aggregation issues that jeopardize their effectiveness. Therefore, TiO_2_ nanoparticles (nps), which become superhydrophilic under UV light, function well as photosensitizer. Subsequently, another study established the use of nanoscale TiO_2_ as a redox coating of in implants [9]. Titanium and its alloys are considered the most promising materials for implants due to their superior properties, which fulfill the specifications of implantation technologies better than other metallic materials, such as stainless steel, CrCo alloys, and tantalum [10]. The growth and the volume of the bone surrounding the implant material are the major factors for successful implant treatment, minimizing infection or rejection [11]. TiO_2_ nanomaterials with tailored porosity have already been developed as an alternative orthopedic implantation material as they support cell adhesion, viability, growth, and differentiation, which are favorable in bone tissue growth and biological implant fixation [12]. Moreover, to minimize the risk of device-related infections, implants are usually coated with TiO_2_ nanotubes, which under UV irradiation, generate reactive oxygen species (ROS), resulting in the disinfection ability [13].

One of the most vital contributions of nanotechnology is the development of novel modes of drug delivery. Ideal drug delivery systems encompass two elements, that is, the control over drug release and the ability to target specific locations in order to reduce systemic toxicity and undesirable side effects. Porous TiO_2_ has shown tremendous ability to sustain a concentration of drugs within the therapeutic window for a convenient timespan to significantly improve the remedy for several diseases, including cancer.

TiO_2_ nanomaterials are often used as photosensitizers or as carriers for the delivery of photosensitizing agents, which enhances therapeutic efficacy by increasing the photothermal conversion efficiency and by an accumulation of photosensitizers in tumor sites. ROS-related cancer therapeutics such as photodynamic therapy, sonodynamic therapy, and chemical dynamic therapy showed great potential to significantly enhance the precision and efficacy of cancer therapeutics [14]. Neoplastic cells containing TiO_2_ nps undergo oxidative degeneration upon light irradiation under the influence of generated ROS and, therefore, these nps are considered as a potent photosensitizer in anticancer photodynamic therapy and the photodynamic inactivation of antibiotic-resistant bacteria [15].

TiO_2_ nanostructures such as nanotubes and nanowires have been utilized in photoelectrochemical sensing for the rapid and precise identification of biological analytes at low concentrations, useful for clinical diagnosis. These nanostructures have been employed for sensing humidity, oxygen, and hydrogen, inclusive of their use as a matrix for immobilizing enzymes for maintaining their biocatalytic activity for a longer duration [16]. Chen et al. describe the use of TiO_2_ as a molecular sieve by designing flower-like microspheres consisting of a magnetic Fe_3_O_4_ core and a hierarchical mesoporous and macroporous TiO_2_ shell for the selective and rapid capture of peptides from human serum and urine samples [17]. Many studies have been published on using TiO_2_ nanotubes as photoelectrochemical glucose sensors for health purposes [18–20].

The present review focuses on contemporary research of TiO_2_ nanoparticles and their clinical applications, including their usage as an implant material, antimicrobial agent, drug delivery vehicle, photothermal therapeutic tool, and antivenom. In addition, the intriguing physical and chemical properties of titania nanomaterials that affect their biocompatibility are also discussed. The advancement of this novel inorganic nanomaterial in theranostic nanomedicine might lead to an era of technology that can be used in real-world clinical settings.

## Review

### Effect of physicochemical properties of TiO_2_ on biocompatibility

Biocompatibility is considered as one of the most important features for a material to be used in the biomedical area. In particular, an appropriate beneficial response should be generated with as low as possible undesirable local or systemic effects in the recipient. To improve the biological performance, TiO_2_ nanomaterials are often processed, surface-functionalized, or post-synthetically modified by adding various surfactants or dopants or organic molecules. The size of the nanomaterial also determines the type of immune response elicited by the body (endocytosis/cellular uptake) [21]. Xu et al. reported that the size of the pores is an essential parameter regarding the hydrophilic or hydrophobic nature of a material as water can percolate more easily inside wider pores than inside smaller pores [22]. Synthesized TiO_2_ is often covered partially with a layer of hydroxy groups that imparts a negative charge to the surface, making them hydrophilic with a small contact angle, which is reported to be favorable for biomedical applications. Likewise, Gatoo et al. proposed that amorphous titania materials are hydrophilic due to the presence of a higher concentration of hydroxy groups upon their surface and the high polarity of the O–Ti–O bond [23]. The surface hydroxy groups can react with water molecules. The thus formed hydrogen bonds account for a good wettability. An annealing temperature below 450 °C still retains the hydrophilic behavior because of the combined crystalline phase (anatase and rutile), but above that temperature, the reduction of the number of hydroxy groups elicits hydrophobicity [24]. The primary physical properties of titanium dioxide that contribute to its biocompatibility are high corrosion resistance, the thermodynamic state at low physiological pH values, the isoelectric point of 5–6, the low ion formation tendency in aqueous environments, and a high strength-to-weight ratio. Moreover, titanium is somewhat negatively charged at physiological pH values because of the formation of a passive oxide layer, and its dielectric constant is equivalent to that of water [25].

The specific energy structure of TiO_2_ is responsible for its photocatalytic activity. Upon UV irradiation, the electrons in the valence band get excited to the conduction band, leading to the formation of electron–hole pairs and the generation of ROS. Subsequently, the generated holes (h^+^) convert water/hydroxide molecules to peroxide/hydroxyl radicals by oxidation. The generated free electrons (e^−^) react with molecular oxygen to generate superoxide radicals by reduction. Several factors contribute to the photocatalytic performance of TiO_2_, such as the structural phase (anatase, brookite, or rutile), defects in the lattice, the degree of crystallinity, morphology (nanotubes, nanorods, nanowhiskers, nanoflower, nanotubes, nanobelts, or nanocrystals), and topographical features such as surface area, size (1–100 nm), and uncoordinated surface sites [26]. The photocatalytic nature of titania is greatly explored in antimicrobial studies as well as in photodynamic cancer therapy.

The cytotoxic properties of TiO_2_ are related to differences in phase composition. The anatase phase has a higher toxicity due to its wider bandgap and effectiveness in the generation of ROS [27]. Lower amounts of ROS, which operate as redox signaling messengers, are essential for optimal physiological cell activity, while greater levels result in signaling loss and unspecific damage to cellular macromolecules, contributing to various pathologies [28]. The generation of excessive ROS by TiO_2_ can lead to fibrosis, allergy, even organ failure, and other toxicities in the human body. It was also found that nps smaller than 100 nm produce more ROS due to their higher surface area [29]. Properties of nps such as surface charge density and zeta potential are influential in determining their reactivity, agglomeration properties, interaction with cells, stability in complex media, and adsorption of proteins. The entry of TiO_2_ nps inside the human body could be through inhalation, ingestion of food, skin lesions, and injections [30–31]. The circulatory system then distributes them to different parts of the body. Kreyling et al. studied the biokinetics and clearance of ^48^V-radiolabeled, pure TiO_2_ ([^48^V]TiO_2_NP) anatase nanoparticles by injecting them intravenously into female Wistar rats. The analysis presented higher accumulation in the liver (95.5% after one day), spleen (2.5%), carcass (1%), skeleton (0.7%), and blood (0.4%) while a detectable quantity of nanoparticles was found in all other organs. The [^48^V]TiO_2_NP content in blood decreased 200-fold within one hour, whereas hepato-biliary clearance of [^48^V]TiO_2_NP from the liver and other organs and tissues continued over the period of 28 days [32]. Likewise, the study of MacNicoll et al. has shown that oral administration of 5 mg TiO_2_ nps/kg body weight did not lead to absorption from the gastrointestinal tract into the blood, urine, or other internal organs. Furthermore, human studies revealed that gastrointestinal absorption of TiO_2_ nps into blood and urine was minimal and that the nps are expected to be removed mostly by renal excretion [33].

When a TiO_2_ nanomaterial circulates through the body, certain biomolecules (such as proteins, phospholipids, or DNA contained in biological fluids or present in living cells) get adsorbed onto the surface of it very quickly, which is termed as “protein corona (PC)” formation. This protein corona alters the surface properties and transforms the physical, chemical, and biological characteristics of the nanomaterial. The types and amounts of adsorbed proteins are influenced by certain physiochemical qualities of the nanomaterial, such as the size, shape, charge as well as topography, hydrophilicity, and functional groups that can affect the PC formation. Interestingly, a dynamic aspect that impacts the PC formation is referred to as the “Vroman effect”, a phenomenon where the proteins that are initially associated with nanomaterials get exchanged by a new set of proteins that possess higher affinities for the nanoparticle surface or the corona. Recently, Zhongru Gou et al. investigated the amount and type of cell adhesion-related proteins (such as fibronectin, vitronectin, and laminin) from serum adsorbed on titanium nanotube arrays. Their findings suggest that all the abovementioned proteins got adsorbed on the nanotube surface and that the nanotopography plays an important role in their selective adsorption and maintenance of biological function [34]. It has been reported that the small size of the nanotubes seems to speed up cell adhesion by providing an effective length scale for integrin clustering and focal adhesion development. In this context, Chen et al. employed the adsorption of functional proteins (bone morphogenetic protein 2 and sclerostin antibody) to modify TiO_2_ nanotube arrays to repair bone fractures [35]. The PC alters biodistribution, biological identity and stability, toxicity, and ultimately the fate of TiO_2_ nps [36]. Thus, there is a need to meticulously characterize the nanomaterial properties, emphasizing particle size, crystal structure, and specific surface area, for a reliable prediction of the toxicological behavior of TiO_2_ nanomaterials. A number of recent studies have indicated that nanostructured TiO_2_ is an inert and safe material and could be used in advanced imaging and nanotherapeutics, as depicted in Table 1.

**Table 1:** Summary of the biocompatible nature of various TiO_2_ nanomaterials.

Nanomaterial	Synthesis method	Shape and size	Surface modification	Biocompatibility	Ref.

TiO_2_ nanocrystalline film as light-addressable electrode	sol–gel	mesoporous structure with pore diameters of 50–100 nm	poly-ᴅ-lysine	glia-neuron co-culture were grown fully within two weeks	[37]
titania–chitosan nanocomposites	sol–gel	spherical and irregular morphology of 4.5–10.5 nm	—	hydroxyapatite (HAp) layer formation	[38]
titania coating over stainless steel cardiovascular stents	sol–gel	—	—	growth and proliferation of human umbilical vein endothelial cells	[39]
titania nanotubes (TNTs)	electrochemical anodization	200 nm in diameter	octenidine dihydrochloride (OCT)/poly(lactic-*co*-glycolic acid) (PLGA) was infiltrated into TNTs	OCT/PLGA-TNTs showed bone marrow mesenchymal stem cells (BMSCs) viability and supported cell proliferation	[40]
TiO_2_ nps	sol–gel	pore diameter 2.42 nm, aggregates of nanoparticles 300–400 nm	GABA, sulfate and phosphate ions	useful for intranasal administration and promote brain delivery of antiepileptic drugs to control seizures	[41]
TiO_2_ nps	—	mean size of ca. 15 nm	co-doped with Fe and N	human dermal fibroblasts retain their specific elongated morphology and established numerous focal adhesions	[42]
nanocomposite of TNTs with silver (TNT/Ag)	chemical vapor deposition	tube diameter 30–45 nm	enriched with silver nanograins	biocompatible with L929 fibroblasts	[43]
porous TNTs	anodic oxidation	diameter 300–500 nm, wall thickness 150–300 nm	—	high biocompatibility with L929 murine fibroblasts and photocatalytic activity	[44]
TiO_2_ was used as coating	physical vapor deposition	30.5 nm titania shell thickness	—	increased penetrability of titania-coated nanoparticles through the elastic lamina	[45]
nanocomposite of Ti6Al4V/TNT/HA	atomic layer deposition	diameter of TNT 18–140 nm	hydroxyapatite	proliferation of L929 fibroblasts	[46]
mesoporous TiO_2_ nanobricks (MTNs)	simple mixing	diamond shape, 220 ± 10 nm in width, 250 ± 10 nm in length and ca. 40 nm in thickness; pore size of ca. 4.1 nm	PEG	good biocompatibility with no apparent changes in morphology in hematoxylin and eosin	[47]
reduced graphene/TiO_2_ composites	—	—	—	stromal fibroblast attachment showed commendable compatibility of the sintered nanocomposite	[48]
TNTs loaded with tetracycline (TC) nanoparticles	electrochemical anodization	diameter 100 nm	PLGA-coated TC particles	osteogenic differentiation of mouse pre-osteoblasts and significant antimicrobial activity without cytotoxicity	[49]
TiO_2_ nps	sol–gel and microwave-assisted hydrothermal synthesis	1–13 nm	Ag/Fe	amniotic fluid stem cells are viable, with an active metabolism and are well attached to the substrate	[50]
TiO_2_@AuNPs	microfluidic process	diameters of 232 ± 109 nm	—	TiO_2_@AuNPs were found to be highly biocompatible for human umbilical vein endothelial cells (HUVECs). Their viability was not affected even at higher concentrations of TiO_2_@AuNPs nanocomposite.	[51]
Fe–TiO_2_ nanosystem	solvothermal method and thermal decomposition	nanorod width 10 and length 30 nm	—	high ratio of viable cells for both 2D breast cancer 4T1 cells and 3D intestine organoids	[52]

### Biomedical applications

#### Titania nanomaterials as implant materials

Implanting is a challenging aspect of medical science since the implant materials are kept inside the body permanently or for a longer time. Moreover, implant materials are often treated as harmful foreign materials and rejected by the human body through immune reactions [53–54]. For an optimum result, a biomaterial needs to be compatible with its physiological environment (such as bone or other tissues). The fusion of living cells with the TiO_2_ layer of the implant occurs in such a strong manner that they can only be separated by fracture and this stable fixation was termed as osseointegration by Brånemark [55]. The oxide layer of TiO_2_ encourages quick and reliable osseointegration and it creates a passivating effect on metal, thereby minimizing corrosion and limiting the release of titanium ions [55]. Furthermore, TiO_2_ has been found to be completely non-toxic, bio-inert, with high fatigue limit, and resistant to corrosion by all body fluids because of the natural formation of a protective oxide film. These properties makes it a material of choice for implants. Additionally, a thin calcium phosphate coating has been shown to improve the biocompatibility and osteoconductivity of implants [56].

To augment osseointegration and tissue generation, as well as to reduce bacterial accumulation in implants, surface modification is increasingly gaining attention. Dental implants have been modified with drug-releasing TiO_2_ nanotubes to overcome the infection caused by the presence of persistent oral pathogenic microbial biofilms [57]. Their nanometer-sized roughness and surface chemistry play a significant role in the interaction between proteins and cells and the material surface. It has also been shown that hydroxyapatite and calcium phosphate mimics the chemical composition of natural bone. Thus, with the use of these components in the coating, TiO_2_ implants have demonstrated enhanced osseointegration [58]. Additionally, drug release kinetics and duration from titania nanotubes (TNTs) can be controlled by modifying nanotube dimensions, surface chemistry, or by a polymer coating on the TNT implant surface through dip coating or plasma polymerization. Losic and co-workers have developed a well-designed controllable drug delivery system by functionalizing 3-aminopropyltriethoxysilane (APTES) on TNTs and found that the drug loading capacity was improved by 30–36 wt % in comparison with unmodified TNTs. Intriguingly, the hydrophilic nature of APTES was favorable for augmenting better attachment of drug molecules, and the drug release profile was extended to more than 15 days by minimizing the burst release effect [59].

**Figure 2:**
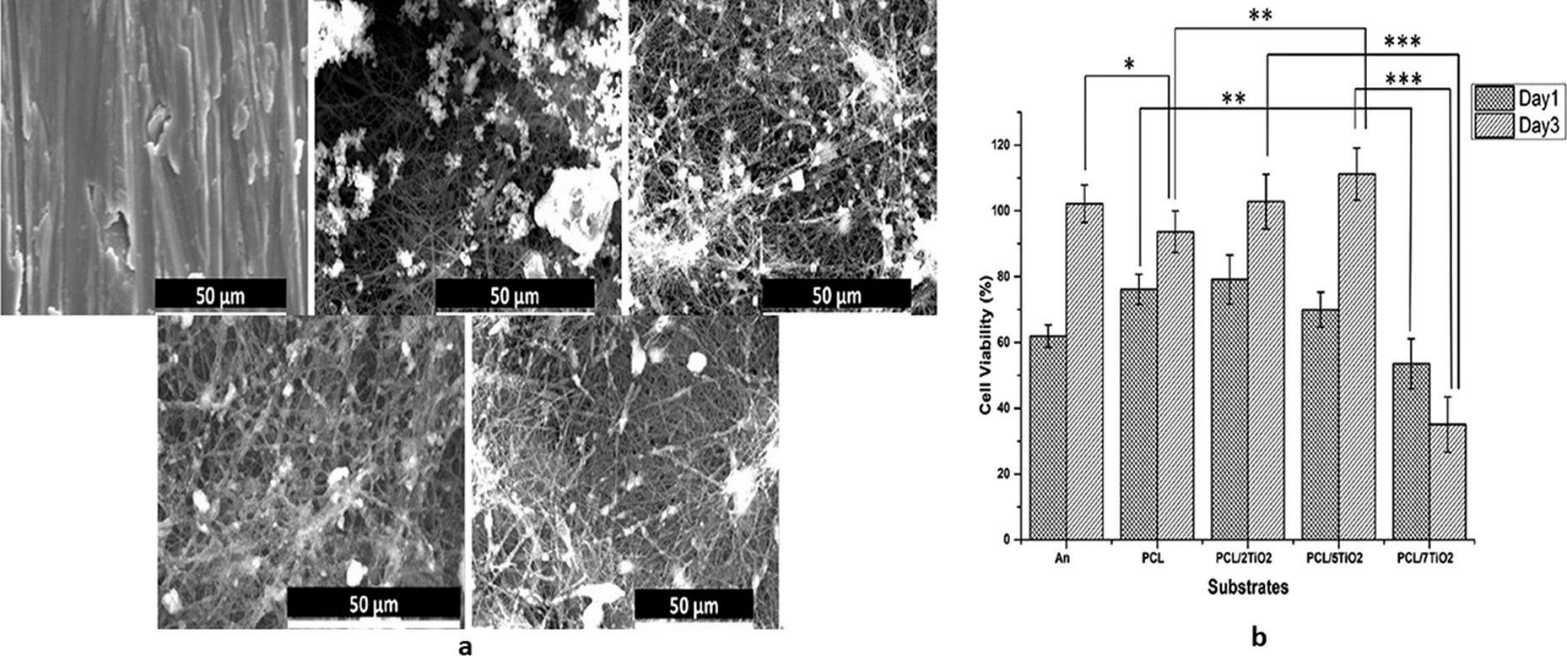
SEM images of (a) annealed Ti, (b) PCL, (c) PCL with 2 wt % TiO_2_, (d) PCL with 5 wt % TiO_2_, and (e) PCL with 7 wt % TiO_2_ after immersing in simulated body fluid for 21 days. (f) Viability of hFOB cells on the five corresponding samples cultured on day 1 and day 3 (*p* < 0.05) (Figure 2 was adapted from [60] (© 2018 A. S. K. Kiran et al., published by MDPI, distributed under the terms of the Creative Commons Attribution 4.0 International License, https://creativecommons.org/licenses/by/4.0).

Polycaprolactone is a semi-crystalline biodegradable polymer used as a drug carrier, packaging material, and 3D scaffold for bone tissue engineering. However, it is hydrophobic and poor cell adhesion has been reported. In a study of Kiran et al., TiO_2_ nanoparticles (0, 2, 5, and 7 wt %) were suspended in polycaprolactone forming a polymer/ceramic hybrid composite (PCL/TiO_2_), which was then used as a coating over biomedical grade commercial pure titanium (cpTi). Thereafter, human osteoblast-like cell lines (hFOB) were cultured over annealed Ti, PCL, and PCL/TiO_2_ (2, 5, and 7 wt % TiO_2_) scaffolds. SEM images of the cell morphologies are shown in Figure 2. The addition of TiO_2_ nanoparticles enhances the wettability and surface area, thus favoring adhesion and proliferation of hFOB. Their results also showed a noticeable reduction in cell viability with a higher percent of TiO_2_ (7 wt %). An antibacterial study of these fabricated structures implied that a minimum of 5 wt % concentration of TiO_2_ is sufficient for achieving the desired antibacterial potential. Thus, the optimized TiO_2_ nanoparticle concentration of the PCL/5TiO_2_ sample exhibited improved biological and antibacterial properties for bone tissue engineering, thereby improving the properties of orthopedic devices [60].

Ko et al. found that titanium covered with a double layer of gold nps (GNP_2_) presented good osseointegration [61]. In another recent study, TiO_2_ nanotubes (TNT) were grown on the surface of medical-grade titanium alloy and then coated with silver nps (Ag nps) to improve the antimicrobial properties of the implants. Moreover, to avoid direct contact of Ag nps with human tissue, the material was covered with a nanoscale hydroxyapatite (nHA) coating and its efficacy was compared to the material without nHA coating. Interestingly, both materials showed antibacterial properties against *Staphylococcus aureus*, but the nHA-coated material was found to be more biocompatible [62]. Non-steroidal anti-inflammatory drugs (e.g., quercetin, ibuprofen, dexamethasone, aspirin, indomethacin) have been successfully loaded and eluted locally from TNTs in vitro in titania-based implants. Surface modifications such as biopolymer coating, polymeric micelle encapsulation, and periodic tailoring of TNTs are employed for delayed/controlled release of anti-inflammatory drugs. Chemical intercalation of the drugs inside the TNTs and the subsequent triggered release are other strategies applied for slow and controlled release [63]. Likewise, gelatin nps, along with the antibiotic vancomycin, were also used to improve the titania implant properties, and the material showed significant antibacterial activity against *Staphylococcus aureus* with sustained release of vancomycin [64].

Furthermore, the biological activity of TiO_2_ nanowires, nanofibres, and nanoneedles, was investigated and compared to that of Ti6Al4V, which is typically utilized in implants. Results revealed that more fibroblast cells proliferated on all specimens of nanofibers as well as on the nanowires arrays when the incubation period was increased; however, this behavior was not observed in the case of nanoneedles [65]. Surface charges of the nanomaterials influence cell adhesion, and cells adhere to hydrophilic surfaces more easily compared to hydrophobic surfaces [66]. Additionally, different phases of TiO_2_ affect the biological properties of the material. For example, the anatase phase absorbs more hydroxy and phosphate ions than the rutile phase in body fluids, supporting the deposition of apatite. A titania nanotube array (anatase) showed increased cell adhesion, proliferation, and differentiation [67].

Titanium heart valves are also very compatible and compete with regular tissue valves [68]. In addition, titanium nitride (TiN) coating has been licensed by the FDA to be used in titanium alloy components for enhancing durability and corrosion resistance in surgical steel, orthodontics, hip prostheses, and cardiovascular biomaterials [69]. A titania/glass ceramic (TiGC) scaffold was fabricated and coated with alginate, gelatin, and chitosan to enhance strength and durability [70]. In another strategy to improve the bioactivity of titania scaffolds, alkaline phosphatase (ALP) was functionalized onto 3D TiO_2_ scaffolds based on a simple dip-coating method. ALP catalyzes the hydrolysis of organic phosphate that contributes to hydroxyapatite (HA) formation and bone matrix mineralization [71]. Likewise, nanophase titania/poly(lactic-*co*-glycolic acid) (PLGA) composites have been designed that showed greater osteoblast adhesion compared to plain PLGA [72].

In vivo tissue engineering (TE) holds tremendous potential in regenerative medicine because of the utilization of the endogenous stem cells of the host or tissue-specific progenitor cells at the injury site. Akermanite is a bioceramic that has received significant attention because, after implantation, it can release Ca, Si, and Mg ions, which enhances adhesion, proliferation, and differentiation of the osteoblasts. However, the low fracture toughness and brittleness of akermanite have limited its use in load-bearing sites of bone tissue. To strengthen the mechanical properties nanoscale titania (nano-TiO_2_) was distributed into the ceramic matrix. A remarkable improvement in the mechanical properties was observed after the incorporation of 5 wt of nano-TiO_2_ and a bone-like apatite structure was formed in simulated body fluid (SBF), which supported cell attachment and growth, showing the potential for bone TE applications [73]. Human gingival fibroblasts (HGFs) are the main connective tissue cells that secrete the collagen-rich extracellular matrix (ECM) for generating soft tissues that bind with the implants. Wang and co-workers reported that the super hydrophilic nanotubular structure of hydrogenated TiO_2_ prepared by anodic oxidation and thermal hydrogenation significantly increases early HGF adhesion, migration, and ECM secretion [74]. The aforementioned studies suggest that nanostructured titania offers additional benefits for the successful and long-term retention of the implants.

#### Titania nanomaterials as antimicrobial agents

The treatment of bacterial infections with antibiotics is widespread. Antibiotics are proven to be highly efficient, but their uncontrolled use has led to the emergence of antibiotic-resistant species that do not respond to any existing drug. Even though new classes of antibiotics are constantly being developed, resistance to any class of antibiotics has been observed, and multiple mechanisms of resistance to each type of antimicrobial agent have been discovered. Hence, to counter drug resistance, efficient bactericidal materials are needed, and nps have been identified as a promising solution for the abovementioned issue [75]. TiO_2_ is considered as a valuable antimicrobial agent due to its photocatalytic activity and self-cleaning properties. Several factors might affect the physicochemical properties of TiO_2_ nps. Crystal structure and shape are the most critical factors responsible for their antimicrobial property [76]. TiO_2_ has selective spectral absorption in the UV region above 3.2 eV for anatase and 3.0 eV for the rutile phase. The absorbed UV light creates electron–hole pairs that migrate to the surface, causing a redox reaction and leading to ROS formation [77]. Since energy levels are not available for TiO_2_ nps to facilitate convenient recombination electrons and holes, the electrons and holes live long enough for a continuous ROS generation on the surface, which is a highly demanded feature of TiO_2_ nps for the eradication of surface microorganisms [78]. Some studies showed that anatase could produce ^•^OH radicals in a photocatalytic reaction, as a result of which anatase has been found to have the highest antimicrobial activity among all crystal structures of TiO_2_ [79]. The mechanisms of titania-induced biocidal activity are mostly by an oxidative attack on the outer/inner cell membrane of the microorganism, as well as alterations of coenzyme A-dependent enzyme activities and DNA damaging via hydroxyl radicals [80]. Furthermore, DNA is sensitive to oxidative damage. In particular, OH* produced by a Fenton reaction attacks the sugar–phosphate backbone leading to the strand break [81]. Interestingly, due to the efficacy of TiO_2_ nps to kill even desiccation-resistant microbes, their value has increased in the food, cosmetic, and drug industries. Recently, glass surfaces coated with silver and TiO_2_ nps showed promising results against bacteria *S. aureus* (Gram positive) and *E. coli* (Gram negative) as compared to the standard glass surface [82].

Another recent study stated that hollow, calcined TiO_2_ nanospheres (CSTiO_2_), synthesized by the combination of electrospinning and atomic layer deposition, have high antimicrobial activity against multidrug-resistant bacteria such as *S. aureus* strains compared to commercial TiO_2_ nps [83]. TiO_2_ supported on silica nanospheres was checked for its antibacterial activity against *E. coli,* and the result demonstrated a more effective growth inhibition than that of commercial TiO_2_-P25 under ultraviolet and visible light [84]. Copper is well known for its antimicrobial properties, and it is considered a potent candidate for modifying TiO_2_ by photodeposition or radiolytic reduction. The prepared material exhibited antibacterial and antifungal properties under UV, visible and solar irradiation, and even in darkness [85]. Intriguingly, an enhanced antimicrobial activity of TiO_2_ nps was reported in a study where the addition of the nontoxic inorganic salt potassium iodide to TiO_2_ (P25) excited by UVA expanded its killing properties of bacteria and fungi up to sixfold [86].

It is well established that, during implantation, if bacteria get in along with implanted parts, they can grow and reproduce inside the body. *Staphylococcus* is the most common bacterial species in this case, and its ability to adhere to the implant materials and promote the formation of a biofilm is the most critical feature of its pathogenicity. Once the biofilm is formed, even routine antibiotic administration is not sufficient. Infection can occur in the blood, bone, or soft tissue such as heart or skin [87]. It often ends with a chronic infection, which is a challenging health care issue and a leading cause of death worldwide. Various reports have concluded that TiO_2_ nps are an effective system for biofilm inhibition and treatment [88–90]. The size of the nps impacts the diffusion into the extracellular polymeric substance matrix, with diameters up to 130 nm demonstrating deep penetration into biofilms. Moreover, positively charged nps exert greater biofilm penetration over anionic or uncharged equivalents. TiO_2_ nps have been presented as an antifungal biofilm agent against *Candida albicans* on the surfaces of biomedical implants [91]. In this context, Dworniczek et al. reported that europium-doped and sulfated anatase TiO_2_ results in the effective photocatalytic inactivation of *Enterococcus* biofilms [92]. Shabib and his colleagues published an interesting study on the synthesis of TiO_2_ nps from the root extract of *W. somnifera* and examined its broad-spectrum antibiofilm potential against *E. coli, Pseudomonas aeruginosa, methicillin-resistant S. aureus, Listeria monocytogenes, Serratia marcescens*, and *Candida albicans*. The result showed that intracellular ROS generation by TiO_2_ nps inhibited and destroyed biofilms of the abovementioned bacterial and fungal species. Furthermore, because some studies have shown that the pro-oncogenic properties of biofilms formed by invasive pathogenic bacteria can support and initiate cancer growth, the cytotoxicity of *W. somnifera*-synthesized TiO_2_ nps was tested against the human hepatic cancer cell line HepG2 and a concentration-dependent decrease in cell viability of HepG2 cells was discovered [93]. Thabet et al. also showed the antifungal efficiency of commercial TiO_2_ nanospheres against *Saccharomyces cerevisiae*, *Botrytis cinerea*, *Candida krusei*, and *Rhodotorula glutini*. Their study revealed over 99% inactivation of *S. cerevisiae* and *C. krusei* and ca. 90% inactivation of *R. glutinis* within the first 5 h, which raised up to more than 99% following a 20 h incubation [94]. According to a recent study, nanocomposites could be highly effective in the removal of biofilm and killing of pathogenic bacteria in comparison to pure TiO_2_ nps without harming healthy human cells. In this context, Baig et al. demonstrated the disinfecting properties of copper oxide/titanium dioxide nanocomposites against biofilm-forming and methicillin-resistant strains of *Staphylococcus aureus* and *Pseudomonas aeruginosa* [95].

**Figure 3:**
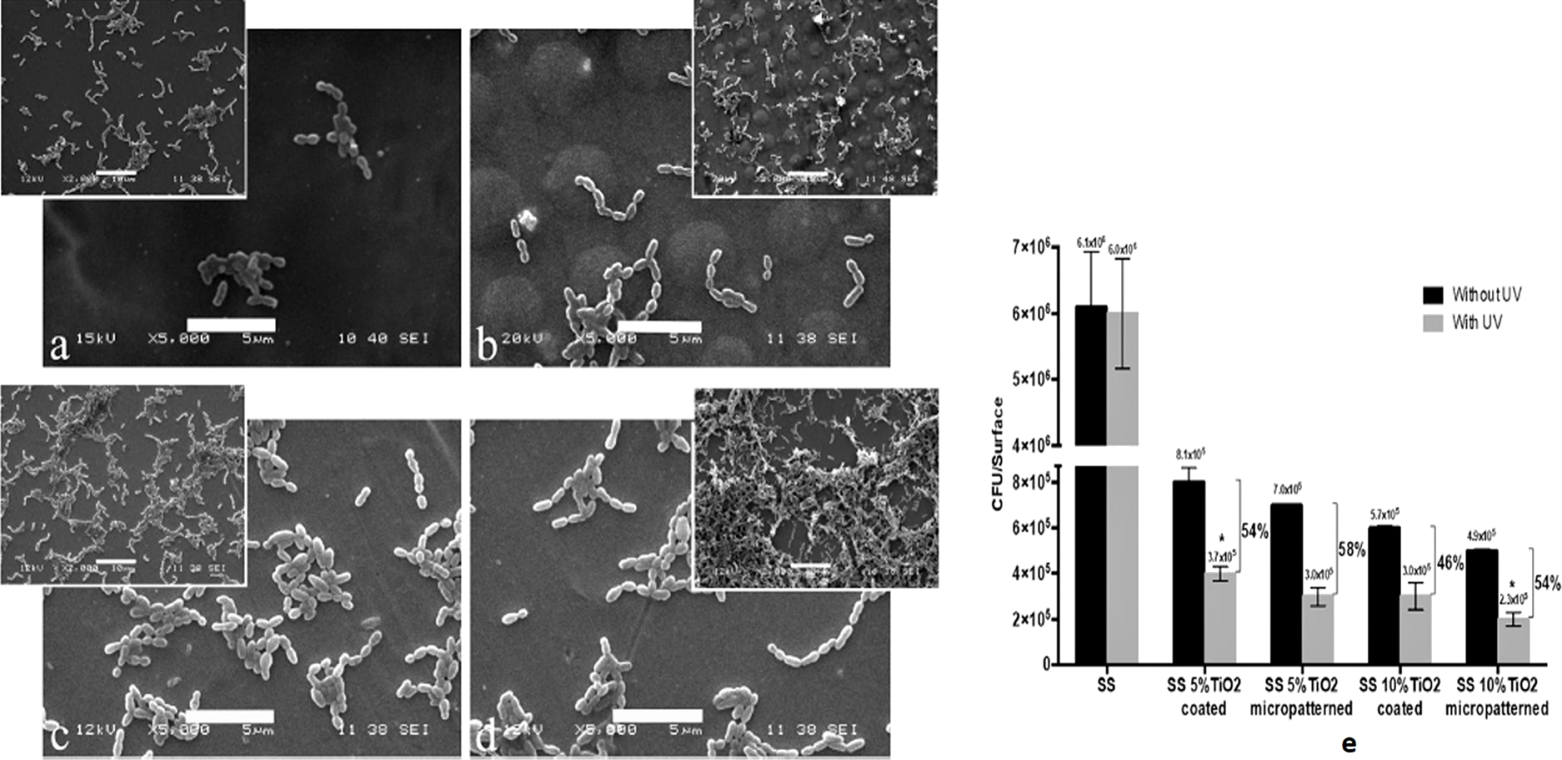
SEM images of the bacterial colonization on (a) coated SS-TiO_2_, (b) micropatterned SS-TiO_2_, (c) polished SS, and (d) unexposed polished SS. The scale is 5 µm. Inserts show interaction at 2000×. The scale in the inserts is 10 µm. (e) Viable adhered bacteria on coated SS-TiO_2_ and SS-TiO_2_ micropatterned with different TiO_2_ concentrations after exposure to UV light. The percentages indicate the decrease of viable adhered bacteria due to UV exposure. All samples without UV are statistically significantly different. *Statistically significant difference compared to all other conditions (Figure 3a–e was adapted from [96] (© 2018 S. Arango-Santander et al., published by Springer Nature, distributed under the terms of the Creative Commons Attribution 4.0 International License, https://creativecommons.org/licenses/by/4.0).

In another novel approach, dip pen nanolithography and soft lithography were used to form a micropattern of a silica sol modified with TiO_2_ (5% and 10% concentration), referred to as SS 5% TiO_2_ micropatterned and SS 10% TiO_2_ micropatterned, respectively, on surgical grade stainless steel plates (SS316L). These samples were checked for the adhesion of *Streptococcus mutans* and the results demonstrate a reduction of adhesion of *S. mutans* by 96% in the presence of the TiO_2_ micropatterns (Figure 3) [96].

TiO_2_ nps are examined in clinical research regarding the ability to destroy organic dirt and inhibit the viability of pathogenic bacteria effectively upon irradiation with visible and UV light [97]. To obtain antimicrobial and photocatalytic properties, researchers apply TiO_2_ nanomaterials into polyester fabrics used for orthopedic bandages, plasters, artificial tendons and ligaments, heart valves, artificial kidneys, and surgical gowns and masks. In one study, the antimicrobial properties of polyester fabrics were analyzed after modification with metal-doped titania nps and undoped titania nps. Interestingly, polyester fabric modified by silver-doped TiO_2_ nps showed the best bactericidal property [98]. Huppmann et al. designed an antimicrobial polymer for medical and sanitary applications using TiO_2_ nps as a filler in a medical-grade polypropylene (PP) matrix, which exhibited a surface change and a photocatalytic effect with the effect of killing bacteria. The abovementioned studies indicate that TiO_2_ as a photoactive material is suitable for eliminating biological threats [99]. Among the different strategies for controlling the spread of the newly identified pathogenic human coronavirus SARS-CoV-2 TiO_2_ “self-disinfecting/cleaning” surfaces appear to be a promising approach. In this regard, Khaiboullina et al. noticed that the ROS generated on the surface of nanosized TiO_2_ in the presence of UV radiation could destroy the human coronavirus-NL63 (HCoV-NL63) through oxidative damage, suggesting a potential use to prevent surface transmission of SARS-CoV-2 as well [100].

#### Titania nanomaterials for drug delivery

Oral and intravenous paths are primarily used for drug administration in humans; however, they have certain drawbacks. Immediately after administration, certain drugs may show an effective concentration in the bloodstream. Yet, the concentration may suddenly fall below the effective dose obstructing effective treatment. Another shortcoming of oral drug administration is the inactivation of the medicine (antibiotics, enzymes, drugs, and other therapeutic molecules) in the gastrointestinal tract. These inherent limitations led to the development of nanomedicines as potent drug delivery vehicles approved for medicinal use and treatment of life-threatening diseases. Several types of nps, such as liposomal, polymer-based, terpenoid-based, and dendrimer nps as well as inorganic nanoscale drug carriers are currently used for drug delivery [101]. Almost all of them show higher bioavailability as their uptake mechanism is by absorptive endocytosis, and the slow release of drugs in the blood circulatory system efficiently maintains the level of therapeutic index. The use of nanomaterials has increased nowadays for more specific drug targeting and delivery, slowing down the dissolution rate of drugs, increasing therapeutic efficacy with the minimum dosage, and also by ceasing the premature loss of drugs through rapid clearance. Additionally, the small size of nanomaterials enables them to permeate through biological barriers in the body, such as the blood–brain barrier, the pulmonary system, and through the tight junction of endothelial cells of the skin. The main goal of loading drugs on nanomaterials is the delivery to specific target cells and a reduced toxicity to normal cells of free drug molecules. Surface modification of the nanomaterials with polyethylene glycol (PEG) is reported to be advantageous for multiple reasons, such as inhibition of recognition by the mononuclear phagocytic system, elimination of in vitro toxicity, and prevention of agglomeration [102].

**Figure 4:**
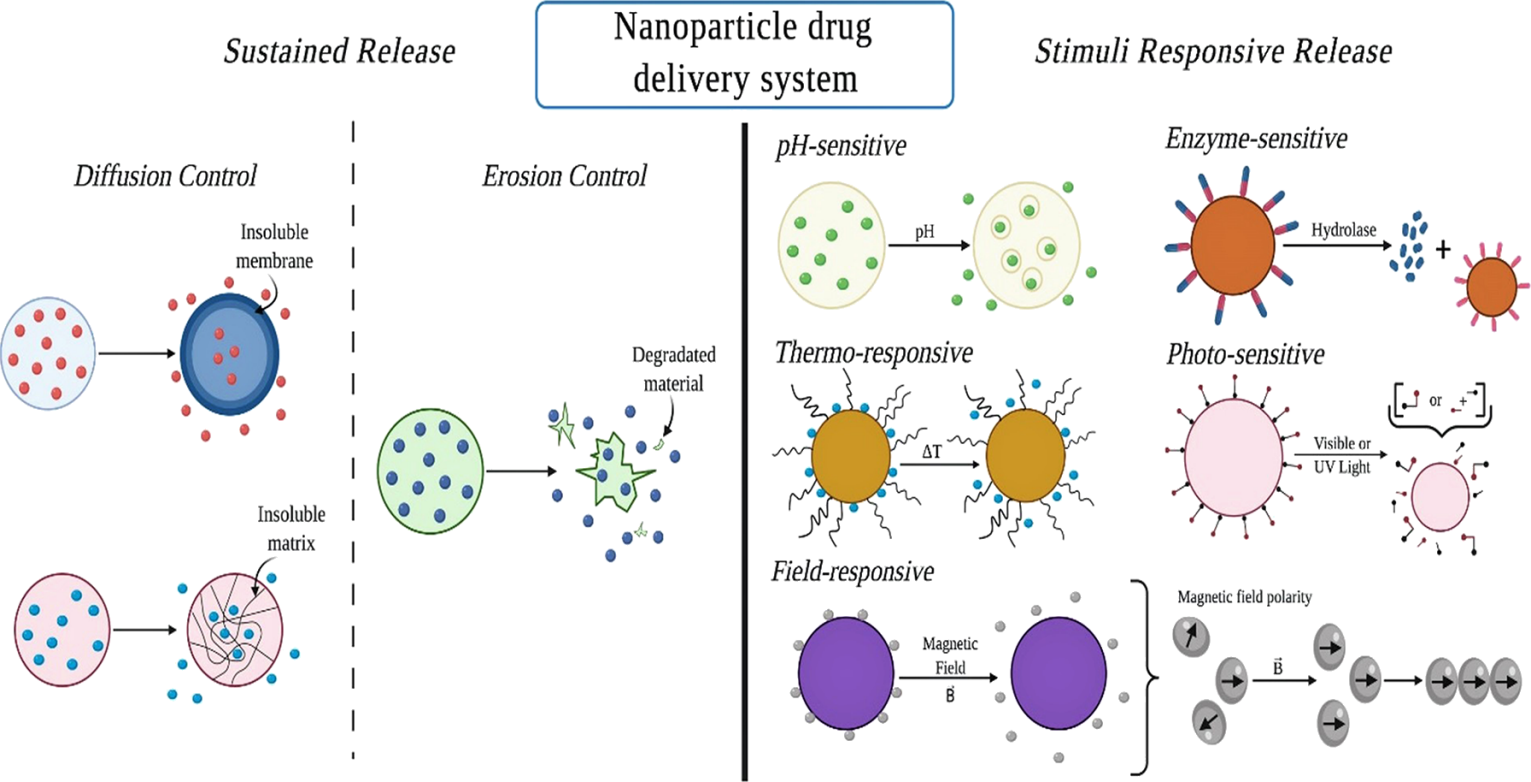
Illustration of the drug release profile of nanomaterials: sustained release and stimuli-responsive release.

Titania nanostructures are capable of loading molecules of various sizes, charges, and solubilities. The immobilization of drugs and their release profile is affected by the size and surface charge distribution of the drug molecule. It has been stated that for the long-term release of pharmaceuticals, nanotubular TiO_2_ can serve as a good candidate as the drug molecule near the surface of the nanotubes will be released quickly, which is called burst release. After that, the release profile will become slower as the drug molecules have to overcome hydrogen bonds and steric hindrance inside the tubular structure. This stage of drug release is known as sustained release. The controlled release of drugs is triggered by various external or internal stimuli. Changes in pH value, redox reactions, and enzyme activity are internal stimuli, while light, magnetic fields, and ultrasound are external stimuli [103]. The drug release profile of different mechanisms is shown in Figure 4. Another parameter that contributes to drug release is the charge of the drug molecule. Due to the presence of hydroxy groups at the surface of TiO_2_, it is supposed to be negatively charged. Consequently, a positively charged molecule will have strong ionic interaction, and the release rate will be slower. This can be described mathematically using Fick’s law, compared to a negatively charged molecule that follows first-order kinetics [104].

**Figure 5:**
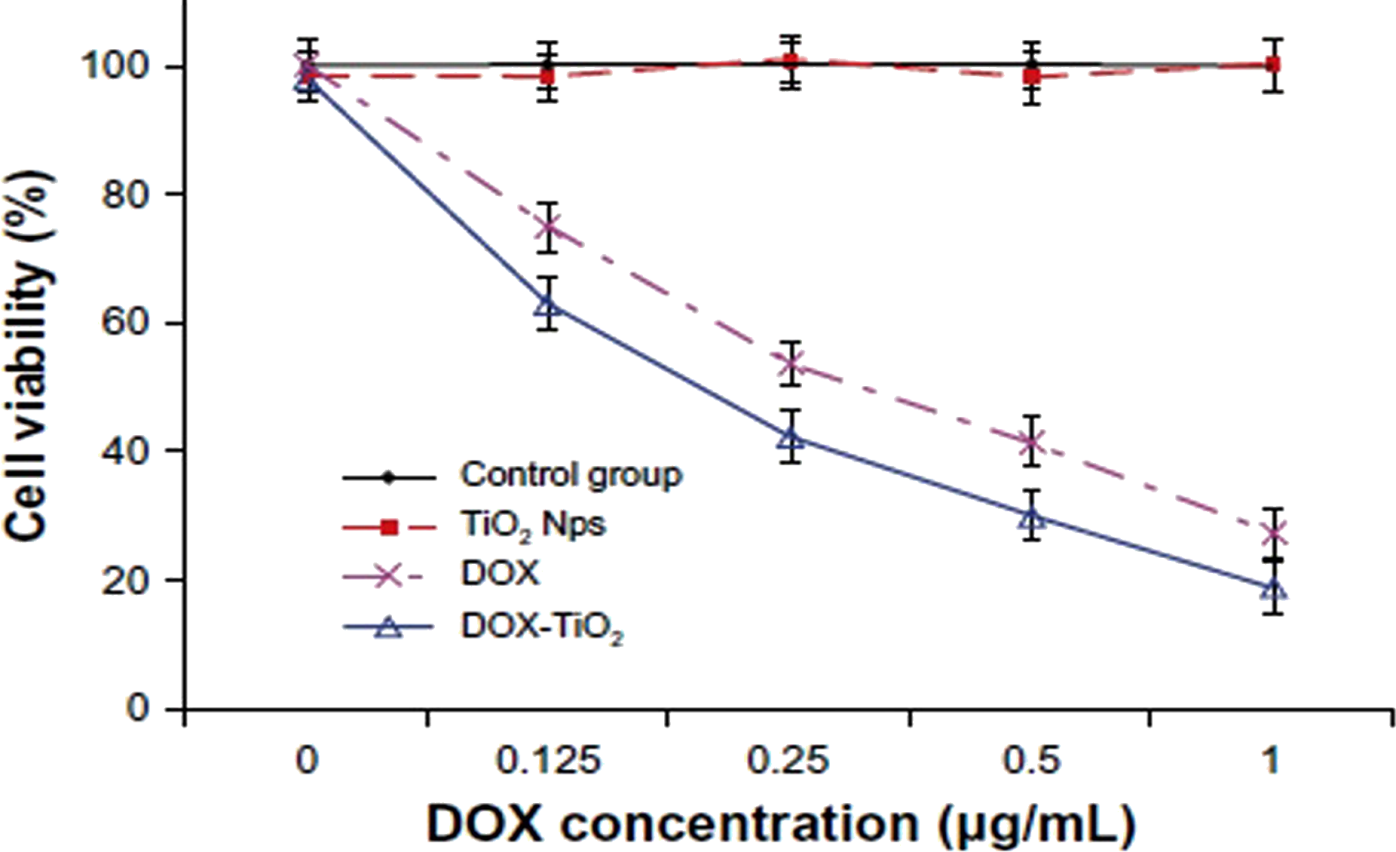
Cytotoxic effect of doxorubicin and DOX-TiO_2_ nanocomposites against human SMMC-7721 hepatocarcinoma cells. Note: data expressed as the mean ± standard deviation (*n* = 3). Cytotoxic effect of doxorubicin and DOX-TiO_2_ nanocomposites against human SMMC-7721 hepatocarcinoma cells. Note: data expressed as the mean ± standard deviation (*n* = 3). (Figure 5 was adapted from [108] “Anticancer efficacy enhancement and attenuation of side effects of doxorubicin with titanium dioxide nanoparticles”, © 2011 Y. Chen et al., published by Dove Medical Press Ltd., distributed under the terms of the Creative Commons Attribution – Non-Commercial (unported, v3.0) License, https://creativecommons.org/licenses/by-nc/3.0/). This content is not subject to CC BY 4.0.

Some recent studies indicated that nanostructured Ti wires might be used in orthopedics as drug-releasing implants and as an alternative delivery system of chemotherapeutic agents to brain tumors [105]. In this context, Jarosz et al. found that the hydrophilic nature of nanoporous TiO_2_ influences the loading and release profile of drug molecules [106]. Moreover, nanoporous TiO_2_ is able to load water-soluble and insoluble drugs and could be useful as an effective drug delivery system [107]. Previously, a drug delivery system based on TiO_2_ nps conjugated with doxorubicin (DOX) was found have an enhanced anti-cancerous effect on human hepatocarcinoma SMMC-7721 cells (Figure 5) by inducing apoptosis in a caspase-dependent manner. Cytotoxicity tests of TiO_2_ nps showed 95% cell viability, ensuring its broad application in biomedicine for cancer therapeutics. Moreover, TiO_2_ nps increases the DOX accumulation in tumor cells while limiting the harmful side effects caused by DOX exposure directly to healthy cells and tissues [108].

In another study, gentamicin was loaded onto nanostructures (nanotubes and nanopores) of a titanium/zirconium alloy nanocomposite (TiZr) coated with chitosan. This composite system followed the Lindner–Lippold mechanism of drug release. The release rate from nanotubes (up to 21 days) was slower than from nanopores, thus opening a new possibility for the targeted treatment of bones and osteomyelitis [109]. These nanoscale drug delivery systems with targeted delivery are rapidly growing and have the potential to revolutionize the efficacy of biomedicine.

#### Titania nanomaterials for phototherapeutic applications

Phototherapy breakthroughs, including photodynamic therapy (PDT) and photothermal therapy (PTT), have established new frontiers in the therapy of cancer and other chronic diseases. The process of inducing cell death using ROS-producing photosensitive materials, followed by irradiation of the target lesion with the light of a particular wavelength, is known as photodynamic therapy (PDT), while PTT is an extension of PDT that causes photon-mediated localized temperature elevation specifically by utilizing infrared radiation, which stimulates hyperthermic physiological responses.

**Figure 6:**
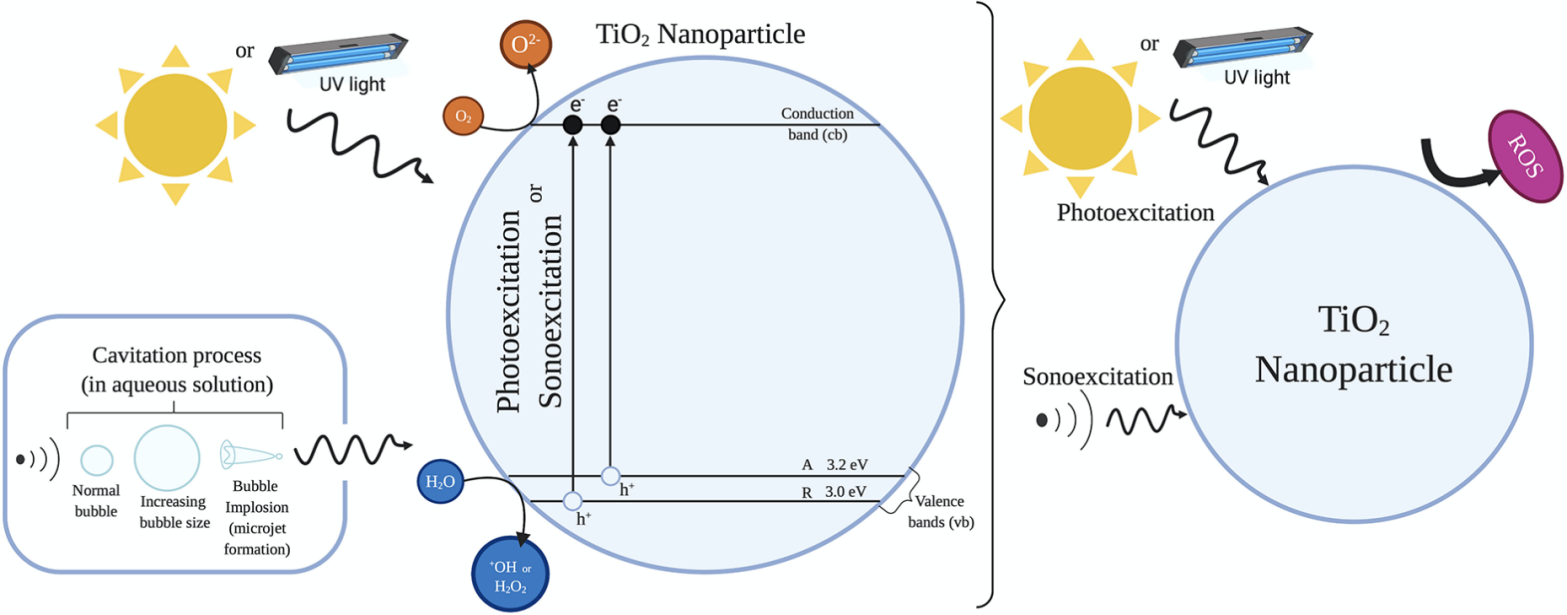
Illustration of ROS generation by TiO_2_ nanomaterials by photosensitization and sonosensitization techniques. (Figure 6 was adapted from [110] (© 2018 J. Bogdan et al., published by Springer Open, distributed under the terms of the Creative Commons Attribution 4.0 International License, (https://creativecommons.org/licenses/by/4.0).

Titania is capable of producing a number of cytotoxic ROS in the presence of sunlight/UV light (e.g., ^•^OH, ^•^O_2_^−^, H_2_O_2_), as illustrated in Figure 6, which may contribute to the death of cancer cells, and has been deemed a suitable candidate for PDT [110]. The principal drawback of using TiO_2_ as photosensitizer is the shallow penetration depth in tissues as it gets activated only by UV light; however, for deep penetration of light into tissues, the wavelength should be in the near-infrared (NIR) window (700–1100 nm) [111].

TiO_2_ nps can be retained in the body for more extended periods of time relative to conventional organic photosensitizers, and they are non-toxic and stable without light irradiation. Thus, TiO_2_ nps activated by NIR light would be an attractive photosensitizing agent for PDT. A team of researchers has synthesized upconversion nps (UCNs) with a thin and continuous layer of TiO_2_ on rare earth nanomaterials and found 50–60% cell destruction when illuminated with NIR light. The results show the penetration of the nanoconstruct into deep tissue tumors, and PEG makes them more biocompatible in conjunction with a strong therapeutic efficacy in vitro as well as in vivo [112]. Photosensitizers utilized in clinical treatments are generally hydrophobic, making them difficult to be used in aqueous systems, thereby reducing their delivery and photosensitizing efficiency. Shah et al. synthesized and modified TiO_2_ nps for safer cancer treatment using PDT. They reported a significant photodynamic effect exhibited by PEGylated undoped-TiO_2_ with 75% killing of HeLa cells at a concentration of 5.5 μg/mL in response to UV or sunlight radiation [113].

**Figure 7:**
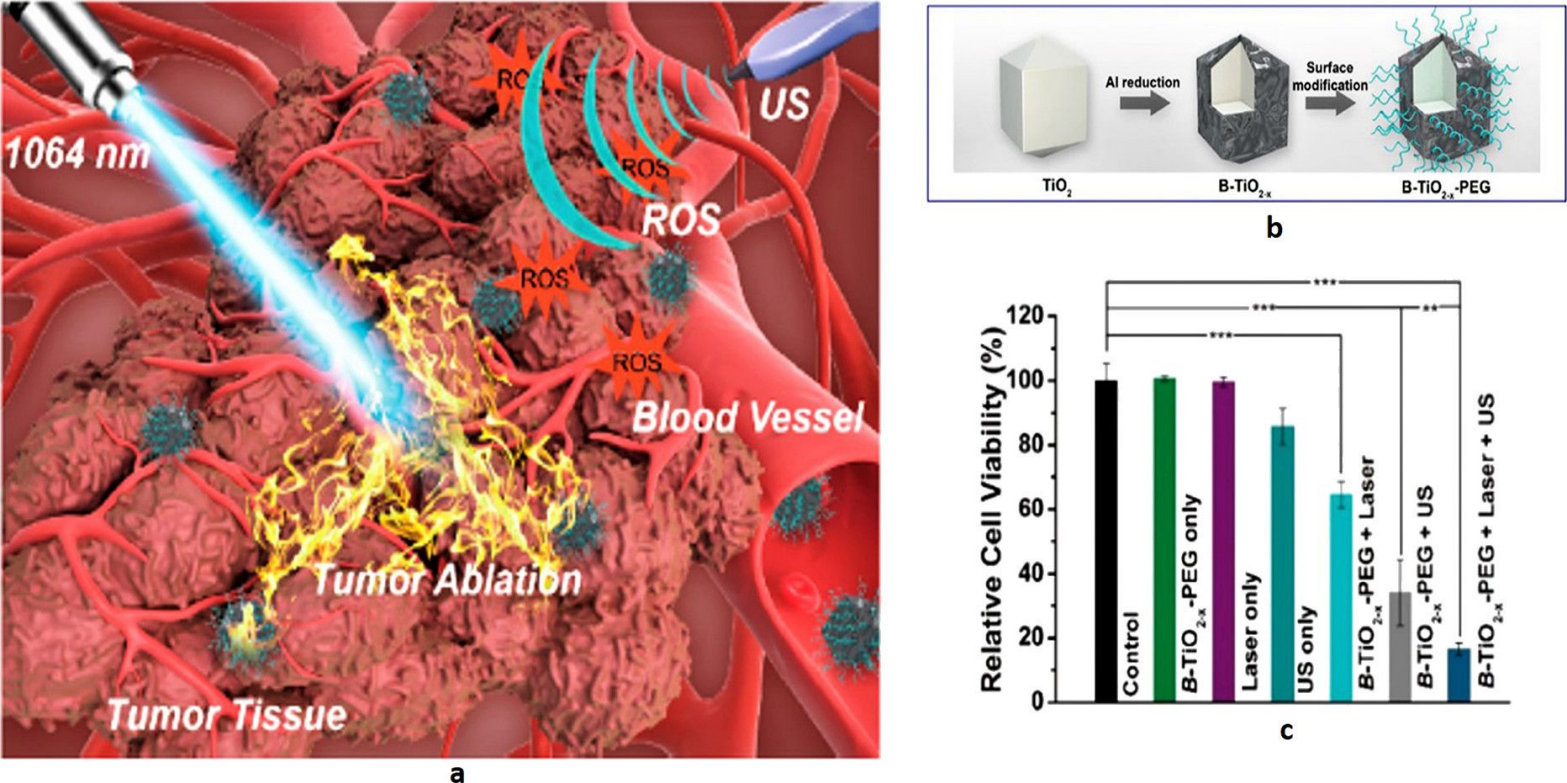
(a) Schematic illustration of synergistic SDT and PTT assisted by B-TiO_2−_*_x_*-PEG for tumor eradication. (b) Schematic of the fabrication of B-TiO_2−_*_x_* by aluminum reduction. (c) Relative cell viability of 4T1 cells after different treatments, including control (without treatment), B-TiO_2−_*_x_*-PEG only, laser only, US irradiation only, B-TiO_2−_*_x_*-PEG combined with laser irradiation, B-TiO_2−_*_x_*-PEG combined with US irradiation, and B-TiO_2−_*_x_*-PEG combined with laser/US co-irradiation (*** denotes *P* < 0.001). (Figure 7 was adapted from [115], Copyright 2011 ACS Publications. This content is not subject to CC BY 4.0).

TiO_2_ and ZnO_2_ are the most effective photosensitizers used in PDT. Yurt et al. conducted experiments in breast and cervical tumors by incorporating zinc phthalocyanine (ZnPc) as photosensitizer into TiO_2_ nps. The result showed a higher cellular uptake of ZnPc-TiO_2_ and an increased PDT efficiency compared ot Zn alone [114]. Since photocatalytic absorption generally occurs at the surface, surface modification acts as the direct route for both bandgap engineering and photoactivity enhancement. One strategy employed was high-pressure and high-temperature hydrogenation, resulting in reduced “black TiO_2_” (B-TiO_2−_*_x_*) nps with a crystalline center and a disordered surface that absorbs light in the visible range. Chen et al. synthesized B-TiO_2−_*_x_* nps by a facile aluminium reduction process and modified its surface with PEG molecules (Figure 7) for high stability under physiological conditions. B-TiO_2−_*_x_*-PEG accumulates in tumor tissue via typical endocytosis processes and functions as nanosonosensitizer as well as photothermal conversion agent. Following ultrasound (US) irradiation, the oxygen-deficient TiO_2−_*_x_* layer with numerous defects facilitates and accelerates the separation of electrons and holes, resulting in a high quantum yield of ROS for tumor eradication. Both in vitro cell-level and systematic in vivo studies of tumor-bearing mouse xenograft demonstrated that upon laser irradiation in the NIR-II window (1064 nm), the tumor temperature reached up to 53.4 °C, inducing complete photothermal eradication of tumor tissue. This study proves the high synergistic efficacy of combined SDT and PTT of B-TiO_2−_*_x_* nps for the complete tumor removal with no evident recurrence, along with relatively high therapeutic biosafety extending their future biomedical application [115].

**Figure 8:**
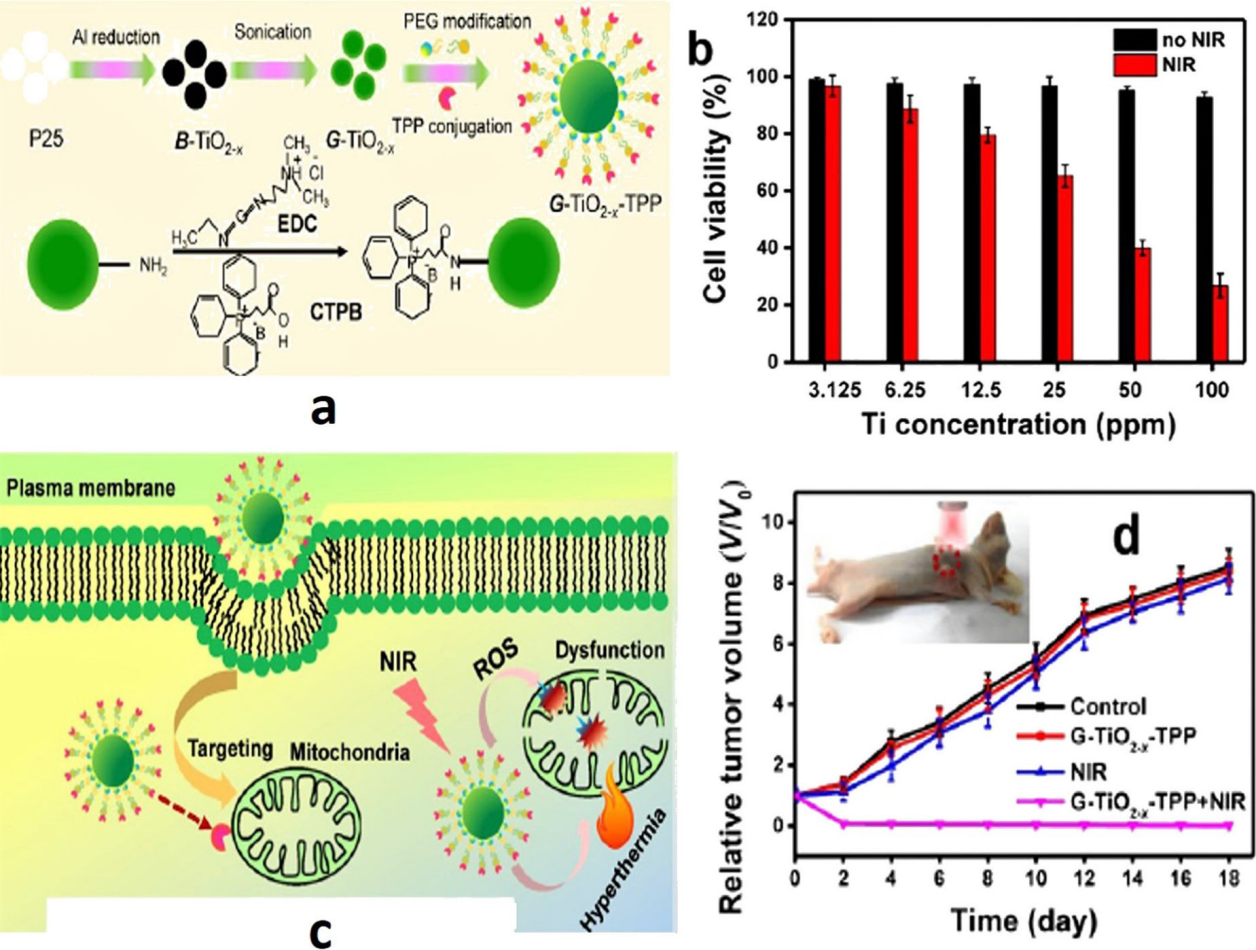
(a) Schematic representation of the preparation and surface modification of green titania (G-TiO_2−_*_x_*) (section A) for (c) mitochondrial-targeted cancer phototherapy. First, black titania (B-TiO_2−_*_x_*) was synthesized from P25 (pristine titania) through an aluminum reduction method. Then G-TiO_2−_*_x_* was prepared from B-TiO_2−_*_x_* by strong ultrasonication. Triphenylphosphonium (TPP) was conjugated to G-TiO_2−_*_x_* (G-TiO_2-x_-TPP) for mitochondria targeting. Under near-infrared (NIR) laser irradiation, G-TiO_2−_*_x_* was able to simultaneously generate reactive oxygen species (ROS) and hyperthermia for photodynamic therapy (PDT) and photothermal therapy (PTT), respectively. (b) In vitro cell viabilities (mean ± SD) of HeLa cells incubated with G-TiO_2−_*_x_* at different Ti concentrations for 24 h without (black bars) and under NIR laser irradiation for 5 min (red bars, 980 nm, 0.72 W·cm^−2^). (d) Tumor growth curves of different groups of tumor-bearing mice. The inset shows a photograph of cancer phototherapy (Figure 8 was adapted from [116] (© 2017 Ivyspring International Publisher, published by Ivyspring International Publisher, distributed under the terms of the Creative Commons Attribution (CC BY-NC) license (https://creativecommons.org/licenses/by-nc/4.0/). This content is not subject to CC BY 4.0.

Recently, PTT and PDT methods that target mitochondria have been developed as new treatment techniques for enhancing therapeutic efficacy. Since mitochondria are the cell key energy centers and are extremely sensitive to heat shock, they contribute to apoptotic cell death by generating ROS. As a result, by lowering the intensity of laser power and dosage, mitochondria-targeted PDT and PTT would provide better results. In the study of Mou and co-workers, a unique type of self-doped green TiO_2_ (G-TiO_2−_*_x_*) was irreversibly produced from black titania (B-TiO_2−_*_x_*) applying intense ultrasonication, as shown schematically in Figure 8A. The G-TiO_2−_*_x_* has been conjugated to triphenylphosphonium (TPP), a lipophilic cation that binds to the mitochondria through insertion into the inner membrane, for precise mitochondria-targeted cancer treatment as presented in Figure 8B. The efficiency of G-TiO_2−_*_x_*-TPP was scrutinized in mice having HeLa tumors, and the results showed excellent mitochondria-targeting potential and strong phototherapeutic efficacy under a single NIR laser irradiation at a far lower power density and low intravenous dosage. Phototoxicity essays of G-TiO_2−_*_x_* on HeLa cells using 3-(4,5-dimethylthiazol-2-yl)-2,5-diphenyltetrazolium bromide (MTT) showed 85% cell viability, confirming that G-TiO_2−_*_x_* itself is non-toxic even at higher concentrations (Figure 8a). Moreover, the tumor growth rate was monitored to analyze the phototherapeutic efficacy, and the results are presented in Figure 8b. The complete elimination of the tumor in the mice treated with G-TiO_2−_*_x_*-TPP+NIR was noticed, whereas mice treated with physiological saline, G-TiO_2−_*_x_*-TPP, or NIR alone exhibited continued tumor growth. The aforementioned results confirm the biocompatibility of this new titania-based nanomaterial and provide new strategies for subcellular organelle-targeted, minimal/non-invasive cancer treatment [116].

Sonodynamic therapy (SDT) generates ROS in deep tissue for the effective treatment of cancer cells. Although conventional ultrasound treatment penetrates deeper in biological tissue and is non-radiative, it has a low tissue attenuation coefficient. Hence, an alternative therapy was developed combining both sonosensitizers and ultrasound techniques. You et al. coated TiO_2_ nps with carboxymethyl dextran (CMD), a hydrophilic polymer to form hydrophilized TiO_2_ nps (HTiO_2_ nps). In vivo mapping revealed enhanced ROS production in ultrasonically treated cells with HTiO_2_ nps, suppressing the growth of tumors [117]. Similarly, when avidin-modified TiO_2_ was used to treat cancer cells, the cancer cells predominantly took up avidin-TiO_2_. Thus the treatment using ultrasound became site-specific. Photodynamic and sonodynamic therapy have the advantages of low cytotoxicity and genotoxicity. Therefore, these therapies are strong alternatives to classical radiotherapy and chemotherapy methods for cancer treatment [15].

#### Titania nanomaterials as antidotes to venom

Snakebites cause significant morbidity and mortality worldwide (around 100,000 deaths annually). The only treatment of snakebites available are antivenoms from immunized animals, which contain specific IgG antibodies. Moreover, the production of conventional antivenoms is challenging. However, with the increasing application of nps in the pharmaceutical sector, researchers have now designed a novel approach to treat snake bites using nps that can bind venom toxins and prevent venom dissemination across the body. In this context, Gomes et al. conjugated gold nps with the antivenomous compound 2-hydroxy-4-methoxy-benzoic acid (HMBA) extracted from the herb Anantamul (*H. indicus*), which was found to be effective in neutralizing all kinds of toxicity generated by the venom of the deadly Russell’s viper [118]. Likewise, silver nps were used to inhibit snake venom toxicity completely [119]. Recently, Chakrabartty et al. found that TiO_2_ nps are able to neutralize the venom-induced lethal activity of *Daboia russelii* and *Naja kaouthia*. Also, hemorrhagic, coagulant, and anticoagulant effects of viper venom were successfully neutralized, as demonstrated by in vitro and in vivo studies. Furthermore, these nanoparticles limited the generation of abnormal body fluid and reduced venom-induced inflammation more efficiently than existing anti-inflammatory drugs such as aspirin and indomethacin [120]. Thus, TiO_2_, along with other nps, can serve as an alternative therapy against snake venom.

## Conclusion

This review comprehensively summarizes the recent approaches related to TiO_2_ nanomaterials in nanomedicine. The distinctive features of TiO_2_ nanomaterials make them the subject of extensive research for a number of applications, such as implants, drug delivery systems, phototherapy, antimicrobial agents, and as antidotes to snake venom. TiO_2_ nanomaterials have admirable potential for bone implants that favor bone cell growth, differentiation, and apatite growth. Furthermore, ROS generation by TiO_2_ nanoscale systems yielding antimicrobial function adds further benefits by reducing implant-related infections. Mesostructures of TiO_2_ were found to be the most efficient systems for efficient drug delivery, compared to microscale and macroscale structures. TiO_2_ has also been reported as an excellent photosensitizer and oxidizing agent for the destruction of tumors and cancer cells by photodynamic and sonodynamic therapy. Additionally, recent studies demonstrated its effectiveness in neutralizing the toxic effects of snake venom and can emerge as a potential antidote to snakebites. This review offers a detailed description of TiO_2_ nanomaterials that were investigated for their ability to mitigate challenges regarding biomedical applications.

Furthermore, ongoing efforts are being implemented to improve nanomaterial synthesis and explore their novel clinical applications. Regarding this, it is crucial to understand the impact of TiO_2_ nanomaterials inside the body and the related toxicity. This review successfully addresses the significant recent biomedical advances of TiO_2_ nanomaterials. In conclusion, TiO_2_ has put forward several innovative platforms that may provide a perspective in clinical development.

## Outlook

For many years, titania has been employed as a colorant in food, cosmetics, and sunscreen. Moreover, Ti-containing metal alloys have been widely utilized in medical fields, because the have a higher biocompatibility than other vastly explored metal oxides such as silica, manganese oxide, and iron oxide nanoparticles. TiO_2_ acts as a DNA intercalator in the cytoplasm, causing DNA damage by generating reactive oxygen species. The explicit cytotoxicity evaluation of TiO_2_, as well as of the incorporated drug molecules, is a major research concern. Moreover, optimal fabrication, in-depth mechanical stability studies, long-term in vivo studies under mechanical load, quantification of local drug release inside the bone microenvironment are further challenges to be addressed for the efficient clinical translation of TiO_2_ implants.

Titania nanomaterials are gaining popularity as antimicrobial agents due to their intrinsic photocatalytic property, which can kill even antibiotic-resistant bacteria in the presence of UV light; however, UV light is not feasible in clinical situations since it poses a hazard to human cells and the significant energy input required is inefficient. In this context, doping of TiO_2_ with copper, graphene, silver, silver and nitrogen, sulfur and cadmium sulfide, or transition metals was found to be effective for bringing the excitation wavelength near to the biological window (650 ≤ λ ≤ 950 nm and 1000 ≤ λ ≤ 1350 nm) by providing secondary energy levels close to TiO_2_ conduction band. Additionally, an appropriate surface modification could be able to enhance the stability of these TiO_2_ nanoparticles in physiological fluids, besides facilitating targeted accumulation in tumor cells/tissues. The optimal innoxious concentration of of light stimuli-responsive TiO_2_ nanomaterials for treating a particular ailment, the treatment time, and the required shift of the excitation wavelength into the NIR region need to be studied thoroughly. Exogenous physical triggers for activating titania nanoparticles in theranostic nanomedicine are unique and highly encouraging; however, the underlying mechanism has still not been fully understood. Furthermore, several publications report therapeutic modalities based on in vitro investigations, but due to a lack of appropriate techniques and the intricate in vivo environment, monitoring and determining the in vivo treatment strategy is extremely difficult. Hence, a rigorous investigation of fundamental properties of TiO_2_ is essential regarding risk assessment and subsequent performance optimization in vivo. Additionally, since significant investment is required for pre-clinical and clinical studies, the majority of current research products fails in clinical translation and commercialization. Therefore, interdisciplinary research should be performed carefully to establish TiO_2_ as the next generation of nanotherapeutics.
